# Validation of mobile eye-tracking as novel and efficient means for differentiating progressive supranuclear palsy from Parkinson's disease

**DOI:** 10.3389/fnbeh.2012.00088

**Published:** 2012-12-13

**Authors:** Svenja Marx, Gesine Respondek, Maria Stamelou, Stefan Dowiasch, Josef Stoll, Frank Bremmer, Wolfgang H. Oertel, Günter U. Höglinger, Wolfgang Einhäuser

**Affiliations:** ^1^Department of Neurophysics, Philipps-UniversityMarburg, Germany; ^2^Department of Neurology, Philipps-UniversityMarburg, Germany; ^3^Department of Neurology, Technische Universität MünchenMunich, Germany; ^4^Sobell Department for Motor Neurosciences and Movement Disorders, Institute of Neurology, University College LondonLondon, UK; ^5^German Center for Neurodegenerative Diseases (DZNE)Munich, Germany; ^6^Center for Interdisciplinary Research, Bielefeld UniversityBielefeld, Germany

**Keywords:** progressive supranuclear palsy, mobile eye-tracking, eye movements, Parkinson's disease, video-oculography

## Abstract

**Background:** The decreased ability to carry out vertical saccades is a key symptom of Progressive Supranuclear Palsy (PSP). Objective measurement devices can help to reliably detect subtle eye movement disturbances to improve sensitivity and specificity of the clinical diagnosis. The present study aims at transferring findings from restricted stationary video-oculography (VOG) to a wearable head-mounted device, which can be readily applied in clinical practice. **Methods:** We investigated the eye movements in 10 possible or probable PSP patients, 11 Parkinson's disease (PD) patients, and 10 age-matched healthy controls (HCs) using a mobile, gaze-driven video camera setup (EyeSeeCam). Ocular movements were analyzed during a standardized fixation protocol and in an unrestricted real-life scenario while walking along a corridor. **Results:** The EyeSeeCam detected prominent impairment of both saccade velocity and amplitude in PSP patients, differentiating them from PD and HCs. Differences were particularly evident for saccades in the vertical plane, and stronger for saccades than for other eye movements. Differences were more pronounced during the standardized protocol than in the real-life scenario. **Conclusions:** Combined analysis of saccade velocity and saccade amplitude during the fixation protocol with the EyeSeeCam provides a simple, rapid (<20 s), and reliable tool to differentiate clinically established PSP patients from PD and HCs. As such, our findings prepare the ground for using wearable eye-tracking in patients with uncertain diagnoses.

## Introduction

Eye movement abnormalities are an essential clinical feature of Progressive Supranuclear Palsy (PSP). Vertical supranuclear gaze palsy or decreased velocities of vertical saccades are a key to the clinical diagnosis of PSP (Litvan et al., 1996). Besides their role as diagnostic signs, eye movement abnormalities disable PSP patients in their daily routine.

Stationary video-oculography (VOG) during head-fixed viewing shows that virtually all forms of eye movements are affected in PSP, with saccadic eye movements being most prominently impaired. Particularly vertical saccades show reduced amplitude and peak velocity when compared to Parkinson's disease (PD) patients and healthy controls (HCs) (Pinkhardt et al., 2008; Chen et al., 2010; Pinkhardt and Kassubek, 2011). Vergence movements and the associated modulation of the linear vestibuloocular reflex are also considerably affected (Chen et al., 2010). The presence of horizontal square wave jerks during attempted fixation of stationary targets is characteristic of PSP (Chen et al., 2010; Otero-Millan et al., 2011). Among these deficits, saccadic peak velocity in the vertical plane shows the sharpest contrast between PSP and PD (Pinkhardt and Kassubek, 2011).

These PSP-specific eye movement abnormalities make clinical investigation of eye movements in patients with Parkinsonian syndromes of great value for differential diagnosis. Correct diagnosis of PSP remains challenging, especially in its early stages (Burn and Lees, 2002). Eye movement abnormalities are not always easy to detect clinically. Particularly, slowing of saccades is a characteristic symptom that can be missed by less experienced neurologists.

Objective measurement devices aid detection of subtle eye movement disturbances. Stationary VOG setups typically require careful calibration, need patient collaboration, and are thus largely impractical for clinical routine. Head-fixed viewing lacks vestibular and other cross-modal information, leaving the relevance of observed eye movement impairment for real-life behavior open. As a first step toward the development of an objective, easy-to-use method for eye movement-based diagnosis, we here tested if recording eye movements with the versatile, head-mounted EyeSeeCam (Brandt et al., 2006; Schneider et al., 2006, 2009) in a brief and simple fixation protocol can differentiate between patients with clinically established PSP as compared to established PD and HCs, and measured gaze in these groups during free behavior. We aimed at establishing the EyeSeeCam's usage in PD and PSP cases and validating its discriminative power between these groups. The parameters established in the present study in clinically established patients shall pave the way for prospective studies with uncertain diagnoses.

## Materials and methods

### Participants

Patients examined in the Department of Neurology of the University of Marburg qualified for participation in the study, if they had clinically possible or probable PSP (Litvan et al., 1996) and were not more advanced than Hoehn and Yahr stage IV (Golbe and Ohman-Strickland, 2007). As defined by the NINDS-SPSP criteria (Litvan et al., 1996), all patients had supranuclear gaze palsy or slowing of vertical saccades at the time of examination, as evidenced by an examiner specialized in the clinical evaluation of ocular movements.

As controls, we included patients with clinically probable PD (Gibb and Lees, 1988) and HCs. HCs were free of neurologic, systemic, or psychiatric diseases, including alcohol or substance abuse, as verified by detailed evaluation of their medical histories and a comprehensive physical examination.

Further exclusion criteria were other neurological disorders, dementia (mini mental status examination <24), presently active psychiatric disorder (e.g., depression or psychosis), structural brain lesion (e.g., brain surgery, stroke with persistent neurological deficit), cataract, or other neuro-ophthalmological disorders leading to functionally relevant impairment. Since glasses cannot be worn with the EyeSeeCam, people requiring visual correction by glasses stronger than ±2 dpt were also excluded.

Before inclusion into the study, participants gave their informed written consent. All procedures conformed to the Declaration of Helsinki and were approved by the local ethics committee (Ethikkommission FB20, Philipps-Universität Marburg).

### Eye and head movement recordings

We used a mobile VOG setup (EyeSeeCam) to record the participants' eye and head movements. Participants accustomed themselves to wearing the device, while the experimental procedure was explained.

**Figure 1:**
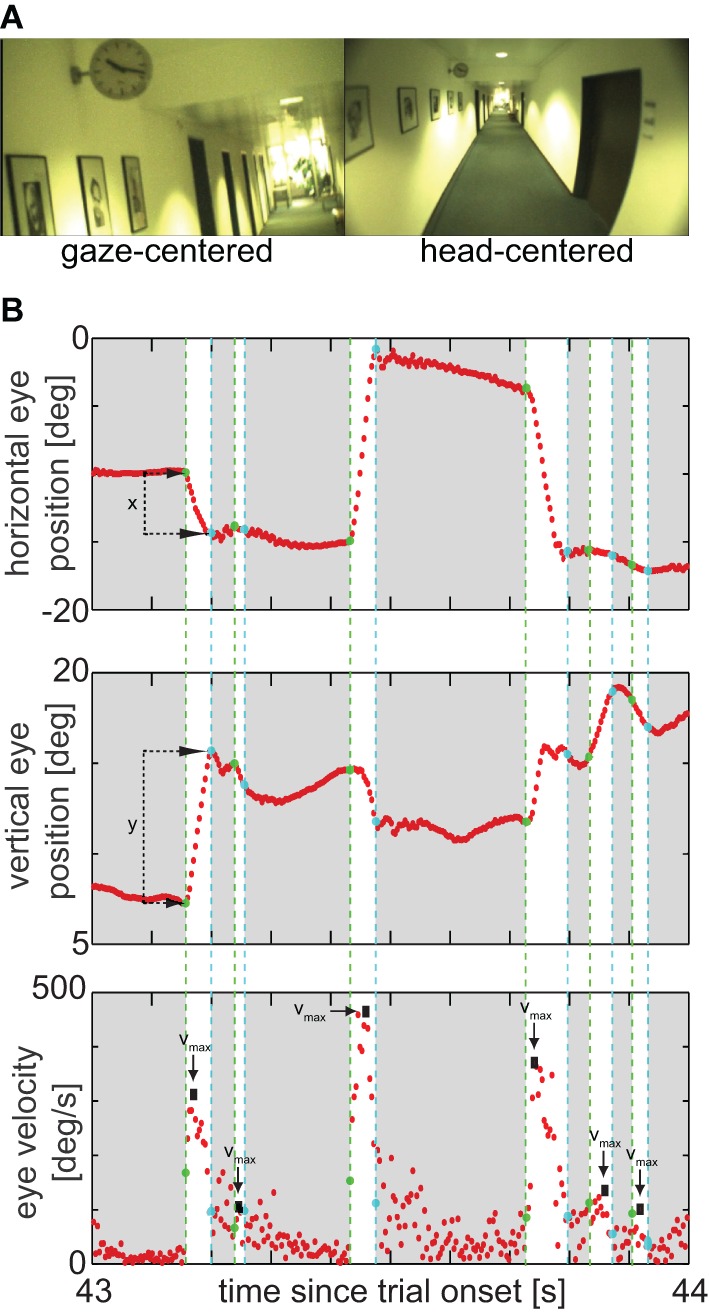
**(A)** Example frame at 43.81 s in the real-life measurement, while a PD patient was looking at the clock; left: gaze camera, right: head camera. The movie of this scene including velocity histograms is shown as supplemental online Movie 1. **(B)** Eye-traces of the scene. Upper panel: Horizontal eye position, indicating the horizontal amplitude of saccades; starting and end points of saccades are marked by green and cyan dashes lines, respectively; durations of saccades are highlighted by a white background. Middle panel: Vertical eye position, indicating the vertical amplitude of the same saccades. Lower panel: Absolute eye velocity, arrows mark saccade peak velocities, used for analysis.

The head-mounted device consists of a head-fixed camera to record the perspective of the head, two high-speed cameras tracking eye-in-head movements, and a camera, which is automatically aligned with the observer's direction of gaze. Gaze- and head-centered videos are recorded at 25 Hz (Figure 1A; Movie 1 in supplementary material); eye movements at 300 Hz.

According to manufacturer's specifications, the spatial resolution of the eye-tracking device is given to 0.02° and the precision (relative error) on the order of 0.1° (“maximal resolution error,” Schneider et al., 2009). The accuracy (absolute error) of the device under ideal conditions is about 0.5° according to specifications, and can substantially worsen if the goggles move relative to the head during prolonged measurements without recalibration. Hence, all analysis reported here only use relative measures, which are unaffected by these drifts, such as velocities and saccade amplitudes.

Being not concerned with absolute gaze orientation (i.e., with high accuracy) comes at the advantage that the device may be operated using an internal (“default”) model of ocular geometry for all participants. In this mode of operation, the mapping from eye measurements on gaze direction does not require a subject-specific calibration, which is in particular beneficial in patients with limited ocular motor control or limited compliance with instructions. Although this sacrifices some precision (depending on the actual head shape compared to the default model), no systematic effect on the measures analyzed here can be expected. For the “fixation protocol” (see below), the default model was used in all participants; for the “real-life measurements” (see below), in those participants, in whom it was possible, the subject-specific model obtained from the fixation protocol was used; for the remainder the default model was also used in real-life measurements. Since the subject-specific adaptation of the model represents a calibration procedure for absolute position, for the real-life measurement, these participants will be referred to as successfully and unsuccessfully calibrated, respectively.

When extracting head movements from the head fixed camera, for the analysis conducted here, the spatial resolution is limited by the pixel width of about 0.3°, even though sub-pixel analysis would be possible in principle. When analysis is based on subsequent frames, this limits the resolution for head movements to about 7.5°/s. While integration over multiple frames would be possible to lower this number, this would come at the cost of lower temporal resolution and thus possibly lumping distinct head movements into one.

#### Fixation protocol

To test the utility of the EyeSeeCam as diagnostic tool, we employed a “fixation protocol.” In addition to being the first experimental part, this protocol also served to refine the EyeSeeCam's calibration for the subsequent real-life experiments by adapting the system's internal eye model to the individual. During the fixation protocol, the participants' heads were unrestrained, but they were asked to avoid head movements as far as possible. They were instructed to move their eyes to look successively at 5 laser dots projected onto a wall straight ahead, a central dot and four at 8.5° in the cardinal directions. An experimenter pointed with a finger at the dot the participant should look at. To give the participant the possibility to self-pace their fixations, presentation of the dots in time was to some degree flexible and not exactly clocked. However, the participant had to look at each dot for 2 s at least once in a time span of approximately 20 s. While this procedure is far less constrained and standardized than usual laboratory measurements, it is still more controlled than the real-life conditions of the present study. This flexible and efficient procedure makes the participation of very severely affected patients possible, presenting a clear advantage over more constrained settings.

#### Real-life behavior

For measuring a large range of gaze behaviors as occurring in real-life situations, we asked participants to perform a series of tasks, while spontaneous eye and head movements were recorded. First, free-exploration behavior was assessed by asking participants to walk along a 50 m corridor. Right before the participant turned around at the end of the corridor, an experimenter laid two paper spots on the floor to assess tracking behavior. Participants were asked to track the dots with their eyes, while walking back toward them. Finally, participants took the elevator and descended one-level to test a situation without active movement in a confined visual environment with subtle vestibular input. Those two PSP and PD patients who were wheelchair-dependent were wheeled throughout the whole procedure by an experimenter instead of actively walking.

The objective of the real-life measurement was to provide a naturalistic set of behaviors, while differences between real-life conditions were not at the focus of the current study. Consequently, all data of real-life measurement were pooled per participant. The real-life measurement lasted less than 10 min per participant.

### Data analysis and statistical evaluation

#### Eye movements

Raw eye-position data were processed offline using MATLAB (Matlab 7.10, The MathWorks, Natick, MA), which was also used for statistical analysis. We calculated eye velocity by differentiation of the horizontal and vertical eye position (Figure 1B). Absolute speed was then calculated as the square root of the sum of the squared horizontal and squared vertical velocity componentss.

All phases faster than 60°/s and lasting longer than 10 ms are referred to as “saccades,” irrespective of whether they were actual saccades or fast phases of reflexive movements (Figure 1B). This threshold is higher than those typically used in laboratory settings, as signals obtained during real-life measurements contain rich eye movement dynamics and are typically noisier than under constrained settings. The conservative choice is, however, consistent with previous research on eye movements in PSP patients: for example, judging from the figures in Pinkhardt et al. (2008), their patients had their 5% percentile of peak saccade velocities around or above 60°/s, meaning that we can still expect to include about 95% of actual saccades with our comparably conservative criterion. Since this criterion could also be employed in practice, it will not affect any conclusion on the discriminability of patient groups. Nonetheless, for the general questions pertaining to eye movement disturbances in PSP and PD, the fact that any threshold must remain arbitrary motivates to add an analysis that does not classify eye movements in saccade/non-saccade, but uses the unclassified (i.e., raw) eye movement data (see below and section “Unclassified Eye Movements”).

Parameters to describe saccades were their direction, peak velocity, amplitude, and duration (Figure 1B). Since peak velocity, saccade amplitude, and duration are typically not independent, the functional relationship of amplitude and peak velocity and of amplitude and saccade duration, the so-called main sequence (Bahill et al., 1975), was also considered for real-life data: we fitted the relation with a power function of the form velocity = *a* × amplitude^b^ or duration = *a* × amplitude^b^, respectively (cf. Garbutt et al., 2003), and considered only the fit parameters *a* and *b* further. Since reliable fits of main sequences require substantial amounts of data, this analysis was only performed for the real-life measurements.

To test whether there is an abundance of one saccade direction in a group, we coarsely classified saccades into equally spaced 45° wedges: horizontal (±22.5° from the horizontal), vertical (±22.5° from the vertical), and oblique (the remaining 4 × 45° = 180°).

For analysis of raw (“unclassified”) eye data (i.e., all data irrespective of whether defined as saccade or not), two-dimensional histograms were used. Each bin of the histograms used for analysis corresponds to a velocity interval of 15°/s in each direction (horizontal and vertical); the central bin ranges from −7.5°/s to +7.5°/s in each direction. The number of samples in each bin is color-coded.

#### Head movements

Head movements were computed from the video of the head-fixed camera at 25 Hz. To obtain head position, the same stationary point of the environment was marked in each video-frame. From this point's position in the camera's field of view relative head orientation in the world was computed. Head velocity was obtained by differentiation of this signal, and was thus independent of this choice of origin. All quantitative analysis was therefore based on velocities. Unlike for eye movements and due to the low spatial and temporal resolution (section “Eye and Head Movement Recordings” top), we could not classify head movements in distinct classes (e.g., fast/slow) with the data at hand. Therefore, all analysis was based on overall velocity distributions for each individual.

#### Statistical analysis

Data are presented as mean ± standard deviation. Statistical evaluation used non-parametric tests for raw eye data, such as amplitude and peak velocity of each saccade (Kruskal–Wallis when three groups were compared and Mann–Whitney-*U*-Test for two groups). To compare these parameters in an exploratory manner across participants, the individual distributions are described by their medians as robust measure (since the distributions are either leptokurtic or prone to outliers). Since these medians can be assumed to follow a normal distribution across participants, the group effects were analyzed by parametric tests; that is, ANOVAs for three group comparisons and two-tailed *t*-tests for two-group comparisons and *post-hoc* tests.

#### Signal-detection-theory measures

For assessing the performance of the classifiers between PSP and PD patients, we performed signal-detection analysis by computing the Receiver-Operating-Characteristic (ROC). The ROC is quantified by its area under the curve (AUC), the cut-off point for maximal specificity and sensitivity, and the corresponding values of specificity and sensitivity. Values are reported such that all values of patients classified as PSP patients are strictly smaller than this cut-off value.

## Results

### Participant characteristics

We investigated 10 PSP patients (6 probable, 4 possible), 11 PD patients and 10 HCs (Table 1). All patients were under treatment in the University Hospital in Marburg. There were no significant differences regarding age, disease duration, and gender between the groups. For all patients Hoehn and Yahr stage was assessed in off-state and, as expected, the stages differed significantly between PSP and PD patients (Table 1).

**Table 1:** **Clinical characteristics of the participants in this study: overview**.

	**PSP**	**PD**	**HC**
*N*	10	11	10
Age (years)	65.9 ± 4.6	65.5 ± 12.7	68.3 ± 9.1
Gender (F/M)	3/7	3/8	6/4
DD (years)	3.9 ± 2.7	6.2 ± 4.7	–
H&Y	3.9 ± 0.4	2.5 ± 0.4	
Wheelchair	2/10	2/11	0/10
Real-life measurement time	304.3 ± 114.4 s	242.2 ± 78.5 s	202.8 ± 35.3 s
**Details**
**Patient ID/gender/age [years]**	**Onset**	**Exam. date**	**H&Y**	**Medication**
PSP01/F/67	2004	08/2010	4	Levodopa
PSP02/M/70	2008	08/2010	3	Amantadine
PSP03/F/63	2007	08/2010	4	Levodopa, Amantadine
PSP04/M/70	2007	08/2010	4	Levodopa, Amantadine, Piribedil
PSP05/F/65	2007	08/2010	3	Amantadine, Rotigotine
PSP06/M/67	2000	08/2010	4	Levodopa
PSP07/M/62	2008	02/2011	4	Levodopa
PSP08/M/74	2005	05/2011	4	Levodopa, Amantadine
PSP09/M/59	2010	10/2011	3	Levodopa
PSP10/M/62	2009	11/2011	3	Levodopa
PD01/M/61	2007	09/2010	2	Rotigotine
PD02/M/75	1995	09/2010	3	Levodopa
PD03/M/75	2007	02/2011	1	Ropinirole
PD04/M/64	2000	07/2011	3	Levodopa, Amantadine, Pramipexole, Rasagiline
PD05/M/67	2007	07/2011	1	Levodopa, Ropinirole, Rasagiline
PD06/M/51	2010	09/2011	2	Levodopa, Rasagiline
PD07/F/62	2007	10/2011	3	Levodopa, Rasagiline, Piribedil
PD08/M/38	2010	10/2011	2	Pramipexole
PD09/M/78	2007	12/2011	3	Levodopa
PD10/F/82	2001	12/2011	3	Levodopa, Amantadine, Ropinirole
PD11/F/68	2000	12/2011	3	Levodopa, Amantadine, Pramipexole
HC01/F/58		08/2010		
HC02/M/71		08/2010		
HC03/F/53		02/2011		
HC04/F/63		02/2011		
HC05/M/73		03/2011		
HC06/F/64		03/2011		
HC07/F/69		03/2011		
HC08/F/74		09/2011		
HC09/M/85		12/2011		
HC10/M/73		12/2011		

PSP, progressive supranuclear palsy; PD, Parkinson's disease; HC, healthy controls; DD, disease duration; H&Y, Hoehn and Yahr Stage. H&Y stage is significantly different between PD and PSP [*t*_(19)_ = *4.12*, *p* < *0.001*]; real-life measurement duration differs significantly between PSP and HC (*p* = *0.02* post-hoc test); all other comparisons do not show a significant difference (*p* > *0.05*).

Eye velocities and relative eye positions (e.g., saccade amplitudes) require only minimal subject–specific adjustment and could thus be measured accurately in all participants. However, individual-specific calibration of absolute eye-position failed in eight PSP and two PD patients as a consequence of their inability to steadily fixate instructed targets over a 2-s integration window. Interestingly, this inability did not primarily result from square-wave jerks, which were robustly observed only in 1 out of the 10 PSP patients under our experimental conditions. As a consequence of the calibration failures for absolute position, all quantitative analysis hereafter is based on relative eye-position and velocities only.

### Saccades

#### Fixation protocol

**Figure 2:**
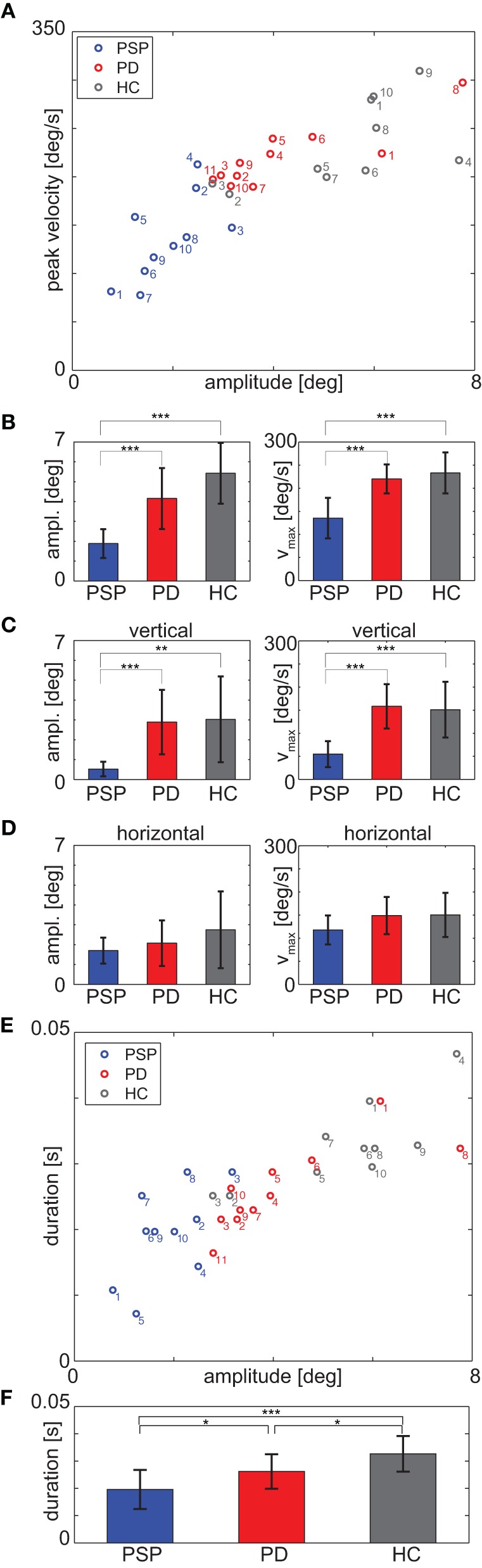
**(A)** Medians of saccade peak velocity and amplitude for each participant during the fixation protocol. **(B)** Mean over participants of median amplitude (left panel) and median peak velocity (right panel) for each group. **(C)** Vertical component and **(D)** horizontal component of the data of panel **(B)**; **(E)** Medians of saccade duration and amplitude for each participant during fixation protocol; note that the duration is discretized due to sampling frequency **(F)**. Mean over participants of median duration. ^*^p < 0.05; ^**^p < 0.01; ^***^p < 0.001.

All participants performed a standard fixation protocol, as described in the “Materials and Methods” section, which was also used for individual calibration refinement. Irrespective of whether this absolute-position calibration was successful or not, these measurements provided a sufficient number of visually-guided saccades to analyze differences between PSP patients and PD patients or HCs (Figure 2).

Averaged median saccadic peak velocity was 135.1 ± 43.8°/s for PSP, 220.1 ± 31.5°/s for PD patients and 233.0 ± 44.4°/s for HCs. A One-Way ANOVA revealed a significant main effect [*F*_(2, 28)_ = 17.81, *p* < 0.001, Figure 2B] and *post-hoc t*-tests showed that PSP patients generated saccades with significantly slower median peak velocity than PD patients [*t*_(19)_ = 5.14, *p* < 0.001] and HCs [*t*_(18)_ = 4.96, *p* < 0.001]. There were also significant differences in the vertical components of saccade peak velocity. Averaged vertical saccade peak velocity was 54.9 ± 28.0°/s for PSP patients, 158.5 ± 47.9°/s for PD patients and 151.1 ± 60.3°/s for HCs [*F*_(2, 28)_ = 14.53, *p* < 0.001; PSP-PD: *t*_(19)_ = 5.83, *p* < 0.001; PSP-HC: *t*_(18)_ = 4.51, *p* < 0.001, Figure 2C].

Saccade amplitudes also differed significantly between groups [*F*_(2, 28)_ = 18.26, *p* < 0.001, PSP-PD: *t*_(19)_ = 4.26, *p* < 0.001, PSP-HC: *t*_(18)_ = 6.60, *p* < 0.001, Figure 2B]. Averaged median amplitudes were 1.88 ± 0.72° for PSP patients, 4.16 ± 1.53° for PD patients and 5.42 ± 1.53° for HCs. Vertical saccade amplitude was 0.52 ± 0.37° for PSP patients, 2.89 ± 1.62° for PD patients and 3.03 ± 2.16° for HCs and thus also differed significantly [*F*_(2, 28)_ = 7.76, *p* = 0.002; PSP-PD: *t*_(19)_ = 4.37, *p* < 0.001; PSP-HC: *t*_(18)_ = 3.57, *p* = 0.002, Figure 2C].

We did not find significant main effects for the horizontal components of peak velocity [*F*_(2, 28)_ = 2.12, *p* = 0.14, ANOVA; Figure 2D] and amplitude [*F*_(2, 28)_ = 1.69, *p* = 0.20, Figure 2D].

The ROC comparing saccade peak velocity of PSP and PD patients showed an AUC of 0.95. Specificity was 11/11 and sensitivity was 9/10 for a cut-off value of 189.8°/s (i.e., all patients having slower peak velocities than this value were classified as PSP) patients. For the comparison of vertical saccade peak velocities, the AUC was 1 and for the cut-off value 111.7°/s, specificity was 11/11 and sensitivity was 10/10. The AUC for the comparison of saccade amplitude was 0.97 with a specificity of 11/11 and a sensitivity of 9/10 for a cut-off value of 2.79°. For the vertical component, AUC was 0.99 and the ROC analysis showed a specificity of 10/11 and a sensitivity of 10/10 for the cut-off value 1.68°.

For completeness, we also analyzed saccade duration in all groups. We found a significant main effect between groups [PSP: 19.6 ± 7.2 ms, PD: 26.2 ± 6.3 ms, HC: 32.7 ± 6.5 ms, *F*_(2, 28)_ = 9.6, *p* < 0.001, see Figures 2E,F]. *Post-hoc t*-test revealed significant differences between all groups [PSP-PD: *t*_(19)_ = 2.25, *p* = 0.037; PSP-HC: *t*_(18)_ = 4.27, *p* < 0.001; PD-HC: *t*_(19)_ = 2.30, *p* = 0.033]. Sensitivity was 7/10 and specificity was 9/11 for the cut-off value 21.6 ms, the AUC was 0.77. These values are much lower than for amplitude and peak velocity and thus less informative where differential diagnosis is concerned. Hence, we hereafter focus most analysis on peak velocity and amplitude.

#### Real-life

**Figure 3:**
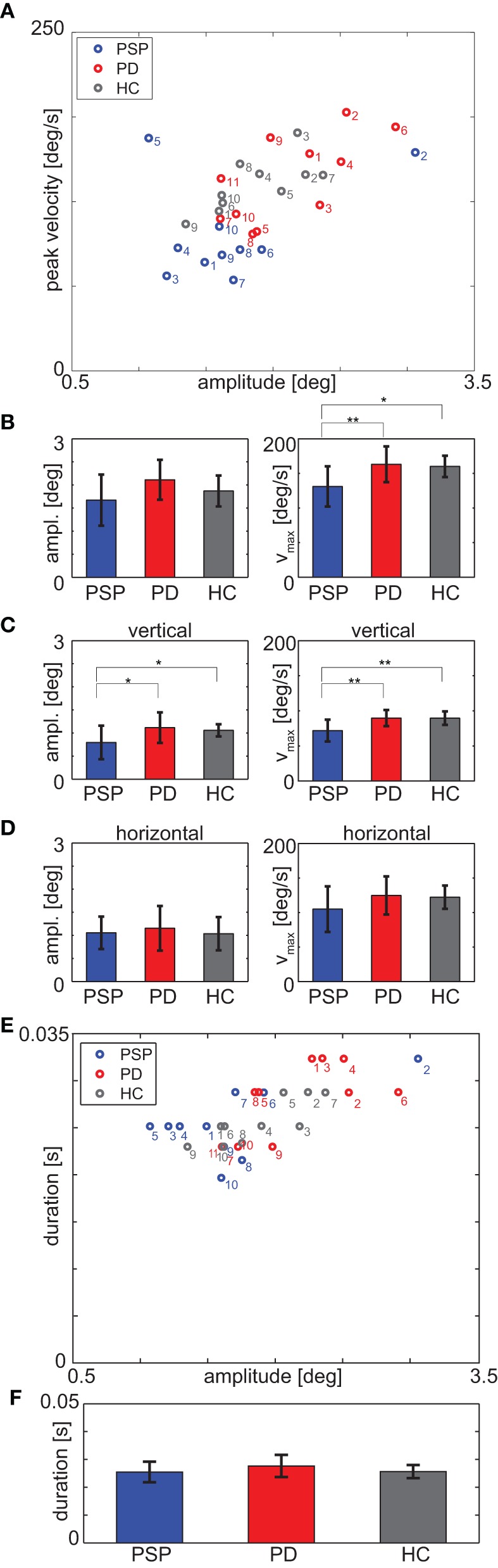
**(A)** Medians of saccade peak velocity and amplitude for each participant during real-life measurement. **(B)** Mean over participants of median amplitude (left panel) and median peak velocity (right panel) for each group. **(C)** Vertical component and **(D)** horizontal component of the data of panel **(B)**. **(E)** Medians of saccade duration and amplitude for each participant during real-life measurement; note that the duration is discretized due to sampling frequency **(F)**. Mean over participants of median duration. ^*^p < 0.05; ^**^p < 0.01.

Since the eye movement impairment in PSP was evident during the fixation protocol, we next analyzed their relevance for real-life situations. Hence, we measured the spontaneous ocular motor behavior in a real-life, minimally restrained scenario, comprising self-paced walking in a corridor, tracking of a stationary target, and taking an elevator. Self-paced walking implies speed differences between participants. ANOVA revealed a significant main effect for differences in real-life measurement duration [*F*_(2, 28)_ = 3.85, *p* = 0.03, Table 1]; the difference was not significant between PSP and PD patients, but for HCs the measurement lasted significantly shorter than for PSP patients [*t*_(18)_ = 2.68, *p* = 0.02]. Aggregating over the whole real-life measurement, we assessed the same parameters as during the fixation protocol (Figure 3).

All groups had the same fraction of vertical [PSP: 24.1% ± 15.4%, PD: 28.9% ± 10.4%, HC: 31.7% ± 7.1%, *F*_(2, 28)_ = 1.14, *p* = 0.33], horizontal [PSP: 21.7% ± 9.0%, PD: 18.5% ± 8.1%, HC: 18.3% ± 5.9%, *F*_(2, 28)_ = 0.64, *p* = 0.53] and oblique [PSP: 54.3% ± 10.1%, PD: 52.6% ± 6.1%, HC: 50.0% ± 3.7%, *F*_(2, 28)_ = 0.92, *p* = 0.41] saccades.

The medians of saccade peak velocity differed significantly between the groups [*F*_(2, 28)_ = 5.47, *p* = 0.01, Figure 3B]. PSP patients' averaged median saccade peak velocity was 131.1 ± 29.0°/s and thus slower than those of PD patients [163.1 ± 25.8°/s; *t*_(19)_ = 2.68, *p* = 0.002] and HCs [160.2 ± 15.4°/s; *t*_(18)_ = 2.80, *p* = 0.01]. The vertical component of saccade peak velocity (PSP: 71.9 ± 15.5°/s, PD: 89.6 ± 11.5°/s, HC: 89.5 ± 9.6°/s) also differed significantly [*F*_(2, 28)_ = 6.88, *p* = 0.004, PSP-PD: *t*_(19)_ = 3.00, *p* = 0.007; PSP-HC: *t*_(18)_ = 3.05, *p* = 0.007, Figure 3C], whereas there was no significant difference between means of the horizontal component of peak velocity [*F*_(2, 28)_ = 1.66, *p* = 0.21, Figure 3D] between groups.

ANOVA did not reveal a significant main effect for saccade amplitude [*F*_(2, 28)_ = 2.55, *p* = 0.10, Figure 3B], but the vertical component of saccade amplitude differed significantly [*F*_(2, 28)_ = 3.46, *p* = 0.045, Figure 3C]; *post-hoc t*-tests revealed that PSP patients' vertical component of saccade amplitude was significantly shorter (0.79 ± 0.36°) than PD patients' [1.12 ± 0.33°; *t*_(19)_ = 2.12, *p* = 0.047] and HCs' [1.06 ± 0.13°; *t*_(18)_ = 2.16, *p* = 0.04]. There was no significant difference between medians of the horizontal components of amplitudes [*F*_(2, 28)_ = 0.25, *p* = 0.78, Figure 3D].

The AUC was 0.84 for peak velocity with a sensitivity of 8/10 and a specificity of 9/11 for the cut-off value 139.9°/s. For vertical peak velocity, the AUC was 0.82 and for a cut-off value of 83.2°/s sensitivity was 7/10 and specificity was 8/11. For analysis of saccade amplitudes, the AUC was 0.80 with a sensitivity of 8/10 and a specificity of 8/11 for a cut-off value of 1.85°. The AUC for comparison of vertical components was 0.75 with a sensitivity of 6/10 and a specificity of 11/11 for the cut-off value 0.69°.

Differences in medians of saccade duration were not significantly different between groups [PSP: 25.5 ± 3.7 ms, PD: 27.6 ± 4.0 ms, HC: 25.6 ± 2.3 ms, *F*_(2, 28)_ = 1.31, *p* = 0.29; see Figures 3E,F].

#### Correlation between fixation protocol and real-life

Median of peak velocity and its vertical component in the fixation protocol and during real-life measurement correlated significantly (*N* = 31, *r* = 0.39, *p* = 0.03; vertical: *r* = 0.50, *p* = 0.004). Thus, the data collected during the fixation protocol not only differentiated between PSP and PD patients, but also in part predicted real-life performance.

#### Main-sequence analysis

**Figure 4:**
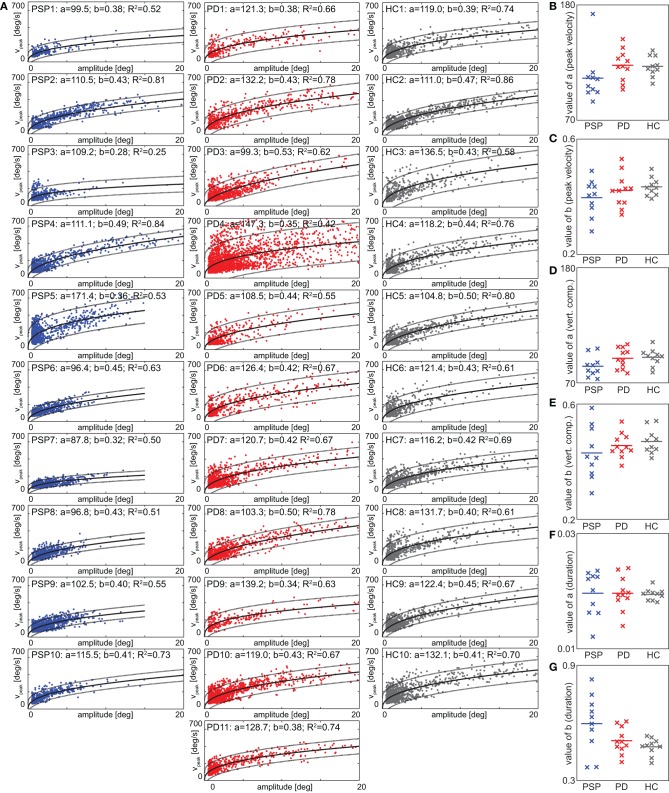
**(A)** Saccade peak velocity plotted against saccade amplitude (“main sequence”) for all individuals. Each data-point corresponds to one saccade (note the cutoff to the bottom and left, given by the thresholds on velocity and duration). Solid black line denotes best fitting power functions (see section “Eye Movements”) in a least-squares sense, dotted lines 5 and 95% confidence intervals. Fit parameters and *R*^2^ are given in the panel headers. **(B,C)** Main-sequence fit parameters of the functional relationship between peak velocity and amplitude. **(D,E)** Main-sequence fit parameters for the functional relationship between vertical component of peak velocity and amplitude. **(F,G)** Main-sequence fit parameters for the functional relationship between saccade duration and amplitude.

Peak velocity and duration were plotted as a function of amplitude for each saccade of every participant. We fitted this main sequence with a power function (Figure 4A) and compared the fit parameters between groups. There were no significant differences between groups with respect to the value of fit parameters *a* [*F*_(2, 28)_ = 1.69, *p* = 0.20, Figure 4B] and *b* [*F*_(2, 28)_ = 1.38, *p* = 0.27, Figure 4C]. There were also no differences between groups in the vertical component of saccades [value of *a*: *F*_(2, 28)_ = 2.54, *p* = 0.097, Figure 4D; value of *b*: *F*_(2, 28)_ = 1.08, *p* = 0.35, Figure 4E] and in the value of the fit parameter *a* of the functional relationship between duration and amplitude [*F*_(2, 28)_ = 0.02, *p* = 0.98, Figure 4F]. There was a significant main effect for the values of *b* in that case [*F*_(2, 28)_ = 4.11, *p* = 0.027, Figure 4G] but *post-hoc t*-tests did not reveal significant differences between PSP and PD patients [*t*_(19)_ = 1.77, *p* = 0.09] or PD patients and HCs [*t*_(19)_ = 1.24, *p* = 0.23]. The only significant difference was found between PSP patients and HCs [*t*_(18)_ = 2.43, *p* = 0.026].

### Unclassified eye movements

**Figure 5:**
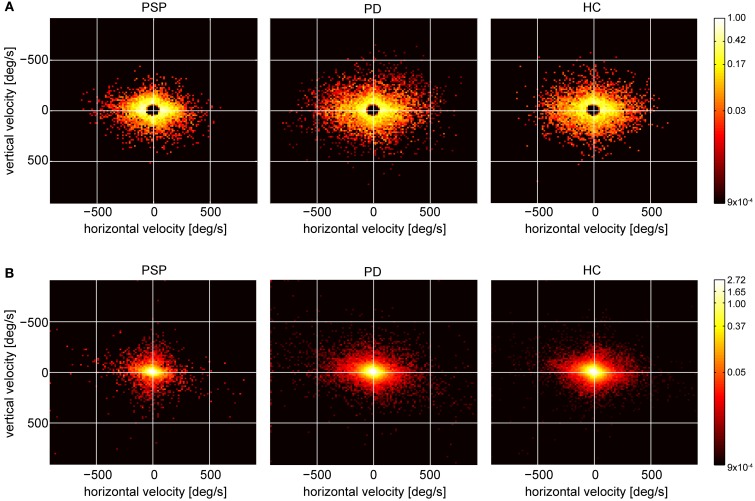
**(A)** Peak velocity histograms of cumulated saccades of all participants in each group (range: −900 to 900°/s in the cardinal directions, bin size: 15° × 15°/s). **(B)** Velocity histograms of raw eye velocities of all cumulated data points of all participants in each group, same range, and bin size as in **(A)**.

Under real-life conditions, fast eye movement phases (saccades), as analyzed above, accounted for only a small amount of the entire measurement time (PSP: 7.6 ± 3.8%, PD: 11.7% ± 7.9%, HC: 10.4% ± 2.8%). To compare saccade-based analysis to all eye movements, we generated 2-dimensional velocity histograms for saccades only (Figure 5A) and for all eye movements (“unclassified movements,” Figure 5B) during the entire real-life measuring time. The histograms show pooled data from all participants of each group, normalized such that each participant contributes with equal weight to the respective histograms. In the distribution of saccade peak velocities (Figure 5A), a preference for horizontal movements is evident in all groups, which is particularly pronounced in PSP patients, reflecting their prominent reduction in vertical peak velocity. Interestingly, this difference between groups was less evident when analyzing all eye movements (Figure 5B). We quantified the spread in each direction by standard deviation. When considering all unclassified eye movements, there were no significant differences among the groups [vertical: *F*_(2, 28)_ = 1.74, *p* = 0.19; horizontal: *F*_(2, 28)_ = 1.86, *p* = 0.18]. When instead considering saccades only (Figure 5A), a picture consistent with the analysis above (section “Real-Life”) emerged: the standard deviation of saccade peak velocities yielded highly significant differences between the groups [vertical: *F*_(2, 28)_ = 8.53, *p* = 0.001; horizontal: *F*_(2, 28)_ = 12.42, *p* < 0.001]. Significant differences appeared between PSP and PD patients [vertical: *t*_(19)_ = 3.38, *p* = 0.003; horizontal: *t*_(19)_ = 4.34, *p* < 0.001] as well as between PSP patients and HCs [vertical: *t*_(18)_ = 3.41, *p* = 0.003; horizontal: *t*_(18)_ = 3.75, *p* = 0.002]. Moreover, when testing analogous measures to those that yielded significant differences and high diagnostic power between patient groups for saccades (Figures 2 and 3), no significant effects were found for the full, unclassified eye movement data. For example, the medians of all velocities were not significantly different between the groups [*F*_(2, 28)_ = 1.01, *p* = 0.38]. Notwithstanding some degree of arbitrariness in the definition of saccade thresholds, this indicates that—at least under our recording conditions—the described effects are best observed in fast movements.

### Head movements

For 26 participants (9 PSP, 7 PD, and 10 HC) we successfully obtained head data during the fixation protocol, for 27 (9 PSP, 9 PD, and 9 HC) during walking along the corridor without target tracking, and for 29 (9 PSP, 10 PD, and 10 HC) while they tracked the stationary target. In the remaining participants, head orientation was not recorded or recording was unsuccessful for technical reasons. We chose to split walking the corridor into periods with tracking and without tracking for head-in-world data considered here, as we expected higher consistency with respect to the overall head movements.

During the fixation protocol, all but one participant deviated less than 2° from their average gaze orientation, 22/26 even less than 1°. Thus, head movements were small and rare, and the median head velocity was below 2°/s in all but one participant. While this implies that participants complied with the instruction to avoid head movements, it also means insufficient movements to obtain robust velocity data.

**Figure 6:**
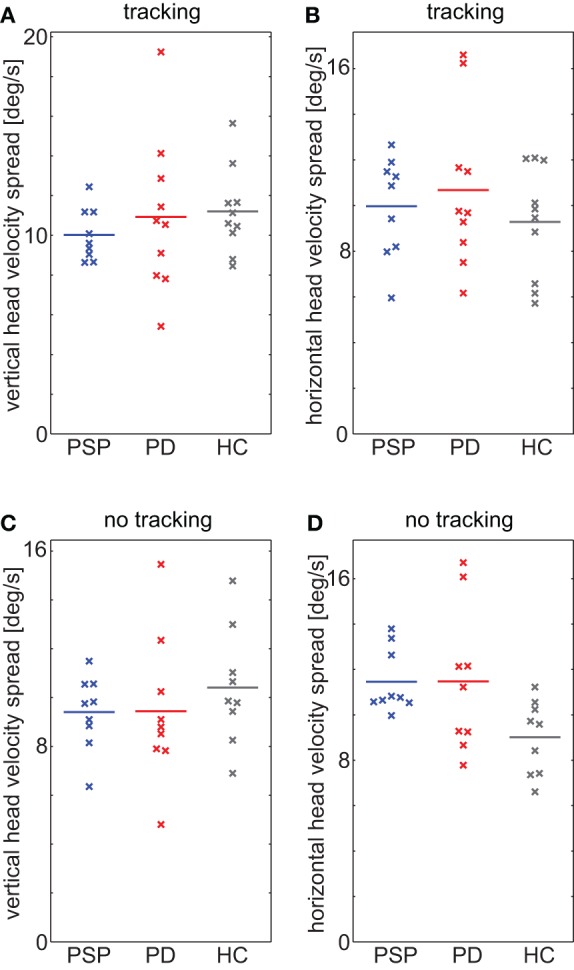
**(A,B)** Spread of head velocity in **(A)** vertical and **(B)** horizontal direction during walking while tracking a stationary target. **(C,D)** Spread of head velocity in **(C)** vertical and **(D)** horizontal direction during walking without specific instruction. Horizontal displacement of datapoints within groups is for improved visibility only.

During tracking, spread (quantified as standard deviations) of head velocities was not significantly different between groups [vertical: *F*_(2, 26)_ = 0.49, *p* = 0.62, Figure 6A; horizontal: *F*_(2, 26)_ = 0.63, *p* = 0.54, Figure 6B]. During walking without tracking, the vertical spread in velocity showed no dependence on group [*F*_(2, 24)_ = 0.51, *p* = 0.61, Figure 6C], either. In contrast, horizontal spread showed a significant group dependence [*F*_(2, 24)_ = 3.67, *p* = 0.04, Figure 6D], indicating that the absence of an effect during tracking, where less participants contributed, was not due to a lack of power. Importantly, this group dependence resulted from a difference between PSP patients and HCs [PSP-HC: *t*_(16)_ = 3.41, *p* = 0.004], but not from a difference between patient groups [PSP-PD: *t*_(16)_ = 0.01, *p* = 0.99] or between PD patients and HCs [PD-HC: *t*_(16)_ = 2.07, *p* = 0.055]. In sum, neither head orientation nor head velocity—to the extent they could be analyzed with the present device—could offer any parameters that might serve to discriminate PSP from PD.

## Discussion

In the present study we used a novel, wearable eye-tracking device to assess gaze behavior in PD, PSP, and HCs. First, we demonstrate that wearable eye-tracking distinguishes PSP from PD with high sensitivity and specificity. Second, we show that these differences in gaze behavior are most prominent for saccades in a brief fixation protocol and less pronounced in activities of daily living.

The observed differences between saccadic peak velocities in the fixation protocol are highly consistent with earlier findings (Pinkhardt and Kassubek, 2011; Boxer et al., 2012). Similarly, the lack of evidence for a difference in peak velocities between the PD group and HCs are in line with previous data (Tanyeri et al., 1989; Pinkhardt and Kassubek, 2011). As such, our data extend earlier findings obtained using visually-guided saccades in standard laboratory setups to wearable eye-tracking, which allows efficient assessment of these parameters in less restrained conditions. Even though many sorts of eye movements are affected by PSP, we focused on saccadic peak velocity and amplitude for reasons of efficiency. Duration of saccades as conceivable alternative turned out to have less diagnostic power, despite some difference in the average. Although amplitude, peak velocity, and duration are not independent, but coupled through the “main sequence,” the functional fit does not provide any additional diagnostic power in real-life data, and requires more data than available from the 20-s fixation protocol, such that amplitude and peak velocity remain as the main diagnostic markers for this rapid assessment. Still, if these two parameters should turn out to be insufficient for differential diagnosis in a patients with clinically uncertain diagnosis, other eye movements like vergence and the linear vestibuloocular reflex can also be measured with the EyeSeeCam.

The comparison between raw data and data filtered for saccades allows three main conclusions. First, it stresses the specifically prominent impairment of the saccade system for PSP patients as compared to other eye movement systems (Chen et al., 2010). Second, it underlines the importance of objective measurement devices to reliably detect potentially subtle eye movement-related disease markers (Bartl et al., 2009). Finally, the comparably mild differences in overall gaze orienting behavior might point to a strategy how the specific deficits may be compensated for and thus offers a promising path for carefully quantifiable therapeutic intervention (Zampieri and Di Fabio, 2008).

The reduced differences in gaze behavior during activities of daily living indicate that patients at least in part compensate for their ocular motor deficits. Analysis of head movements, however, suggests substantial inter-individual differences, indicating that compensation strategies are largely idiosyncratic. Predicting such compensation behaviors and relating them to other parameters, such as disease progression, will be an interesting issue for further research in larger, heterogeneous PSP cohorts. In a longitudinal study, the precise quantification of compensatory behavior might then also aid the efficient monitoring of treatment success. For differential diagnosis, the free exploration paradigm is clearly less valuable, demonstrating the importance of a flexible, but at the same time standardized fixation protocol for clinical use. Nonetheless, the free exploration data may yield important information on compensation mechanisms and the consequences of the disease on everyday life.

In contrast to eye movements, the parameters considered for head movements did not allow a significant dissociation between patient groups under any of the tested tasks. This could be due to the low spatial and temporal resolution of the head movement measurements as compared to eye movement measurements. It is conceivable that with an improved measurement device for head movements, with different instructions or tasks, or when effects on eye-head coordination are measured with sufficient spatial and temporal accuracy and precision, head movements might eventually become useful and could augment a PSP/PD discrimination system. However, with the present technology and based on the tasks used in the present study, eye velocity and amplitude during the fixation protocol present a most promising candidate for dissociating PSP from PD also in subclinical populations.

This study is to be regarded as a first step toward establishing a new method as a diagnostic tool. Prospective studies measuring eye movements of still unclassified patients are needed to prove that subclinical oculomotor disturbances can be detected prior to the establishment of the clinical diagnosis. Also, square wave jerks which are characteristic of PSP patients could only be detected in one PSP patient, even by careful visual inspection of all eye movement traces. While beyond the scope of the present study, the question as to whether their absence from the measured data is a technical limitation or a true effect of the population and condition at hand remains an important issue for future research.

Importantly for a possible application in diagnosis and treatment monitoring, the usage of the wearable eye-tracking device is efficient, requiring less than 20-s for the fixation protocol and virtually no device-specific training. While wearable eye-tracking has recently been suggested as tool in a variety of ocular motor and vestibular conditions (Hayhoe and Ballard, 2005; Schumann et al., 2008), the present study demonstrates that wearable eye-tracking also lends itself for efficient clinical use in the context of more complex syndromes, such as typical and atypical Parkinsonism. Whether or not wearable eye-tracking will allow diagnosis beyond the current gold standard obviously can only be established in a long-term longitudinal prospective study, which will apply the criteria found herein already early during disease, when current clinical criteria are not yet clear cut.

## Conflict of interest statement

The authors declare that the research was conducted in the absence of any commercial or financial relationships that could be construed as a potential conflict of interest.

## Supplementary material

**Movie 1 | Example movies of two participants, PD07 and PSP09, showing a part of the real-life measurement.** Histograms picture eye velocity (left panel, range: −500 to 500°/s in the cardinal directions, bin size for this movie: 5°/s × 5°/s) and head velocity (right panel, range: −60 to 60°/s in the cardinal directions, bin size: 3°/s × 3°/s).
